# The importance of culture in predicting environmental behavior in middle school students on Hawaiʻi Island

**DOI:** 10.1371/journal.pone.0207087

**Published:** 2018-11-12

**Authors:** Rachelle K. Gould, Daniel H. Krymkowski, Nicole M. Ardoin

**Affiliations:** 1 Environmental Program, Rubenstein School of Environment and Natural Resources, University of Vermont, Burlington, Vermont, United States of America; 2 Department of Sociology, University of Vermont, Burlington, Vermont, United States of America; 3 Graduate School of Education and Woods Institute for the Environment, Stanford University, Stanford, California, United States of America; Auburn University, UNITED STATES

## Abstract

Researchers have investigated the factors that influence environmental behavior for decades. Two often-investigated phenomena, connectedness to nature and self-efficacy, often correlate with environmental behavior, yet researchers rarely analyze those correlations along with underlying cultural factors. We suggest that this is a substantial oversight and hypothesize that cultural factors affect environmental behavior, particularly through an interplay with the connectedness to nature and self-efficacy constructs. To test this hypothesis, we surveyed eighth-grade students on the island of Hawaiʻi. The instrument included items to assess connectedness to nature and self-efficacy (both frequently measured in environmental behavior studies) and multiple measures of behavior. Most of the behavior measures are commonly used in studies of environmental behavior, and one was developed in collaboration with local partners to reflect more culturally specific modes of environmental behavior. With those partners, we also developed a construct reflecting the relevance of local culture. We explored the relative influence of the more commonly investigated constructs (connectedness to nature, behavioral variables) along with the newer construct (cultural relevance). We found that, when we took those considerations into account, cultural relevance significantly predicted connectedness to nature, self-efficacy, and a commonly used behavioral measure. Our results thus suggest that many models of environmental behavior may be misspecified when they omit critical culture- and ethnicity-related factors. This may be particularly important in contexts with high cultural, racial, and ethnic diversity or in contexts where mainstream Western environmental approaches are non-dominant. Our results emphasize the importance of addressing ethnicity and culture in environmental thought and action.

## Introduction

Decades of research into how people think about the environment and make decisions regarding their own environmental impact have deepened our understanding of the variables that influence pro-environmental behavior [1,2]. Existing quantitative models of pro-environmental behavior explain different amounts of observed variation in environmental behavior, with R^2^ values ranging from 0.11 to 0.49 [3]. Although ample sociological research demonstrates the central role of social structures, ethnicity, and culture in environmental behavior more generally (e.g. [4,5]), quantitative environmental-behavior models rarely include factors related to culture, which we define as social context, symbolic meanings, and communal history that support and perpetuate shared attitudes, knowledge, and values [3,6]. Like other scholars, we suggest that these two strands of research (first, the individual-level focus derived from the psychological and natural resources perspectives; second, the focus on structure, culture, and ethnicity from the sociological and anthropological perspectives) are both influential in understanding, predicting, and disentangling environmental behavior. Research that draws from both realms explores phenomena such as how community attachment and satisfaction or social context relate to environmental behavior. Examples include studying the pathway to a dependent variable of environmental behavior by considering independent variables such as a measure of social context that incorporates participation in civic activities, volunteering, and face-to-face contact with friends in environmental organizations[7] and connecting validated measures of community attachment to environmentally related action [8]. Thus, quantitative, model-based research has explored relationships between behavior and established constructs such as community attachment and sense of place, and between behavior and novel constructs such as social context. Yet none of this work conceptualizes culture as related to both current social context and community history.

In the natural resource and social psychology literatures that consider factors influencing environmental behavior, two oft-discussed concepts are connection to nature and self-efficacy, which we define and discuss in the following section. Another construct that may be important in understanding relationships is cultural relevance, which we define as the relevance of social context and community history, and which accounts for the role of cultural context in psychological processes [9,10]. Research on the role of culture in psychology and behavior is largely separate from (though occasionally responds to) discussions in the natural resources fields of connections to nature, self-efficacy, and behavior. This psychological work on the centrality of culture to psychology and behavior, based in cross-cultural research and critical cultural perspectives, suggests the vital role of culture in influencing how people act in the world [9,11]. Yet to our knowledge, this work has not explicitly addressed environmental behavior.

Connectedness to nature has been a topic of study for decades [12]. Researchers have used many names, nuances, and measures for related constructs that address humans’ emotional and psychological connections to and sense of personal closeness to (or distance from) nature [12,13]. Researchers use both explicit and implicit measures of the concept (i.e., asking people about their connections to nature using connection-related words (e.g. [14]) and having people visually identify overlap between themselves and nature (e.g. [15]). Studies have demonstrated that connectedness to nature positively correlates with numerous measures of subjective wellbeing, including life satisfaction and positive affect (see[13]). Many studies also explore whether stronger nature connections are associated with greater (i.e., more or “better”) pro-environmental behaviors (both self-reported and observed) (e.g. [16]). The precise pathways proposed differ, but the general assumption is that, when people care more about and feel more connected to nature, they will be less apt to act in ways that harm it. Many–but not all–studies have found evidence supporting that assumption. The complexity of this relationship makes it difficult to incorporate all possible influences (i.e., to control for the many possible confounding variables), which may partly explain these mixed relationships [16–18].

Many behavior theories, while varied in relationships among variables, also include some aspect of self-efficacy, which refers to a person’s perception of his/her ability to take action to achieve a desired outcome [19]. The language of self-efficacy gained prominence with Bandura’s theory of that name [20]. His work relates to the idea of locus of control, a decades-old psychological concept describing one’s self-perceived ability to influence events and outcomes [21,22]. These efficacy-related constructs emphasize the importance of empowerment for motivating behavior: people are more likely to act when they feel effective and empowered, or when they feel they have the ability to undertake an action *and* that those actions will make a difference. These notions of efficacy also represent a core component of the Theory of Planned Behavior [23]. In that theory, the final step is “perceived behavioral control,” or perceived control over the performance of the target behavior. Ajzen [24] recognizes close connections between “perceived behavioral control” and self-efficacy and describes how theorized “perceived control” is comprised of beliefs about both self-efficacy and the controllability of a behavior [24].

Researchers have demonstrated that self-efficacy and locus of control correlate with engaging in pro-environmental behavior in a variety of contexts. Examples include correlations between self-efficacy and recycling [25] and between perception of capability to improve the environment and actions taken to mitigate environmental degradation [26]. Yet other studies have found self-efficacy and related concepts, such as response efficacy, to have a variety of complex relationships with behavior [21,27]. That self-efficacy frequently does not correlate with behavior is one of the most notable challenges the theory faces.

Connection to nature and self-efficacy are far from the only constructs explored in the environmental behavior literature. The scholarly literature on pro-environmental behavior demonstrates that environmental behavior correlates with a variety of factors [28]. Three key elements, or themes, identified in a meta-analysis of 20 years of environmental behavior research are knowledge, behavioral constraints/opportunities, and attitudes and values [3]. Within this complex array of models and frameworks representing influences on environmental behavior, however, the role of cultural diversity is rarely incorporated. For one, the majority of these studies incorporate only small percentages of individuals from racially/ethnically non-dominant backgrounds, or sometimes none at all. And even when study populations reflect some cultural and ethnic variability, that cultural dimension is rarely considered in a nuanced manner as a predictor of or explanatory model in environmental behavior models.

Findings from cultural psychology emphasize the crucial role that cultural context, often operationalized as ethnicity in psychological studies, can play in cognition and decision-making [29]. Relatedly, a growing body of work in cultural and educational psychology finds significant differences between people with indigenous and non-indigenous backgrounds in organization of cognition about the biological world. These include differences in the primacy of ecological relationships and perceptions of connections between humans and nature [30–32]. Most of these differences have been found in adjacent rural populations (e.g., indigenous participants who live on a Native American reservation and European heritage respondents who live in the rural area adjacent to the reservation). That different cultural schemas exist seems clear, but researchers have rarely examined how culture and cultural identity may interact specifically with environmental behavior.

Researchers have identified and explored the relevance of indigenous ways of knowing to environmental issues. A robust body of literature describes how indigenous cultures, knowledge systems, and practices address environmental challenges. This work draws from ecology and ethnobotany (e.g. [33]), history (e.g. [34]), education (e.g. [35]), religious studies (e.g. [36]), sociology (e.g. [37]), and anthropology (e.g. [38]), among others. In Hawaiʻi, places, plants, animals, and their associated practices, have enduring cultural importance [39–41].

In this paper, we consider how perceptions of the relevance of a local culture that is heavily influenced by indigenous perspectives might impact self-reports of environmental attitudes and behavior. We address the question: when an index representing the relevance of a local culture heavily influenced by indigenous perspectives is considered alongside self-efficacy and connection to nature in a predictive model of environmental behavior, what role does culture play? We hypothesize that, especially in a place where cultural identity is of great societal importance, cultural relevance may be more strongly connected to self-reported environmental behavior than the more commonly explored constructs of connection to nature and self-efficacy.

## Methods

### Study site

We undertook this study in a location, Hawaiʻi, where nature and culture are both vital for many residents. In addition, Hawaiʻi is a demographically diverse state [42]. In this context, issues of cultural background and identity are common topics of discussion and social concern [43]. The long-term residence status of the majority of our sample was important: Most of our middle school-aged respondents had lived in the area for a decade or more (i.e., for their entire lives) and, thus, grew up surrounded by Native Hawaiian culture and community.

### Partners and survey instrument development

To address our research questions, we collaborated with local Native Hawaiian community members with decades of experience working on social issues in the area, to develop and implement a survey. Our partners provided an in-school program with intertwined environmental-cultural themes and, after a few years working with students, they desired to understand the ways in which connections to local culture and history might interact with environmental science content in helping students learn, think about, and act in support of the environment. The partners co-developed, provided advice on, and assisted with revising our survey items. This collaboration contributed to the cultural and environmental relevance of our instrument for local eighth graders.

We designed a survey with two types of questions. The first type included commonly used measures of the theoretical constructs we described: connectedness to nature and self-efficacy. The second type included collaboratively developed measures designed to address our research question and hypothesis. This latter type explored students’ connections with local culture and locally relevant environmental behaviors. (The full survey instrument is included in the Supplementary Materials (S1 File).)

We used existing measures for (1) connectedness to nature, and (2) self-efficacy as it relates to environmental issues. Mayer and Frantz’s connectedness to nature scale [14] assesses cognitive and affective dimensions of human relationships with nature [44] and been used in a variety of contexts [16]. We worked with our partners to select a subset of these items most appropriate to the context of this study. To assess self-efficacy, we similarly worked with our partners to select a subset of validated items that Stern, Powell, and Ardoin [45] developed and implemented when studying self-efficacy in a culturally responsive environmental education program in the eastern United States. Reliability measures for the items in both of our indices suggest that all items in each index measured the same construct (see Results).

To measure self-reported pro-environmental behavior, we used three items that our community partners suggested would assess locally relevant actions. Our partners suggested we include an item related to watching clouds as an example of a cultural and pro-environmental behavior advocated by Hawaiian ways of being in the world. This behavior is an example of the type of practice they teach in the program: it combines mindful awareness of environment (closely observing cloud type, location, and movement) with place-specific ecological knowledge (what different cloud phenomena portend in this specific biophysical context). Traditional Hawaiian knowledge undergirds this selection: Many*ʻolelo noʻeau*, or traditional Hawaiian sayings, emphasize the important role that cloud observation plays in awareness of and connection to oneʻs environment, one outcome of which is to predict weather. Relevant *ʻolelo* include: (1) “*Kūkula ka ʻike i ka ʻōpua”* (“Knowledge is set up in the clouds”) [46] (pg. 205); (2) “*He hōʻailona ke ao i ʻike ʻia*” (“Clouds are recognized signs”) (Ibid., pg. 67); (3)“*Aia i ka ʻōpua ke ola*: *he ola nui*, *he ola laulā*, *he ola hohonu*, *he ola kiʻekiʻi*” (“Life is in the clouds: great life, broad life, deep life, elevated life”) (Ibid., pg. 7); and (4) “*Noho no ke kanaka a ka lā mālie*, *kau ka ipu hōkeo a ka lawaiʻa*, *nānā ana i ka ʻōpua*” (“A person waits for a clear day, sets up the gourd that holds the fishermanʻs paraphenalia, and observes the clouds”) (Ibid, pg. 253). We also included items measuring other types of pro-environmental behaviors, turning off the water and picking up litter, that are more often a focus in northern/Western cultures and programs. Our community partners suggested these behaviors because they are commonly encouraged in the area. (With varied wording, researchers commonly use such actions as measures of self-reported pro-environmental behavior.)

To address culturally related attitudes, we worked with our partners to develop items that comprise a “cultural relevance” index. The index includes items related to ancient Hawaiian wisdom, community history, and traditional cultural practices (Table 1). We designed the index to highlight local culture and resonate with respondents from diverse ethnic backgrounds. Hawaiian cultural identity heavily influences local culture, yet local culture also incorporates ideas from the many other ethnic and social backgrounds present in Hawaiʻi today. We intentionally specified “ancient Hawaiian wisdom” in one of the items, but used “my community” in the other two items because the partners felt that this struck an appropriate balance between referring to the island’s deep history and its present-day community. According to our partners, this balance approximates Hawaiian culture as it exists today: a complex melding that highlights indigenous perspectives and incorporates modern influences in the islands. A strong reliability statistic for this index suggests that these items create a coherent construct.

**Table 1:** Descriptive statistics for each index (i.e., summative scale) as well as full text of survey items used in analysis for each individual variable. The items in the summative scales all feature Likert-type responses (possible values range from 1 to 5). Higher values indicate more agreement with the statement. The item regarding trash was reverse coded for consistency.

Summative Scales and Their Components	Mean	Standard Deviation	Observed Range	Cronbach's Alpha
*Cultural Relevance*	*11*.*735*	*1*.*903*	*11*	.*761*
I feel comfortable applying ancient Hawaiian wisdom.	3.816	.791	4	
I believe that understanding the history of my community makes me a stronger person.	3.878	.803	4	
I feel it's important to learn about traditional cultural practices in my community.	4.041	.717	3	
*Connectedness to Nature*	*14*.*469*	*2*.*484*	*12*	.*766*
I think of nature as a family I belong to.	3.816	.751	3	
I often feel a strong connection to nature.	3.612	.808	3	
I feel related to animals and plants.	3.480	.721	3	
I identify strongly with Kona’s shorelines.	3.561	.942	4	
*Self-Efficacy*	*16*.*939*	*2*.*380*	*12*	.*774*
My actions impact the environment.	4.408	.686	3	
I have the power to help protect the environment.	4.040	.896	4	
I can make a change in my community.	4.031	.805	4	
The choices I make today can change my entire life.	4.459	.676	2	
**Demographic and Behavioral Variables**				
Gender(1 = female,0 = male)	.531	.502	1	
Race-Ethnicity(1 = Native Hawaiian,0 = Other)	.449	.500	1	
I pay attention to the way the clouds are moving.	2.786	1.077	4	
I ignore trash when I see it on the shoreline.	2.571	.786	3	
I turn the water off when I’m brushing my teeth.	4.204	1.121	4	

### Data collection

The program met for a total of four days over the school year (August through May). The program, which our community partners developed and managed, combined cultural and environmental elements, such as surveying coral reefs, testing water quality, reenacting Native Hawaiian governmental processes, and participating in construction projects using traditional Hawaiian materials and techniques. During the program, teachers learned alongside students and helped teach aspects of the content, as appropriate.

During development of the research instruments, the lead author and study partners met with all of the teachers, as a group and individually, to discuss the program and the survey. Due to the teachers’ heavy involvement in the study, teachers were sensitive to the importance of standardizing survey delivery, and they therefore followed the same time limit and script when administering the survey.

Stanford University’s Institutional Review Board (IRB) approved this research (Protocol # 22288). The IRB waived the requirement for informed, written consent of parents or legal guardians because this activity was consistent with typical classroom activities and students remained anonymous.

Homeroom teachers administered surveys near the end of the school year (May 2012) to all eighth-grade students. Teachers introduced the survey as related to an educational program in which all students had participated throughout the year. Teachers offered students the option of whether to participate in the research and made clear that a lack of participation would not affect their grade. All students present on the day of sampling chose to participate (n = 110). The teachers provided roughly 15 minutes of class time for students to complete the survey.

### Statistical analysis

Our dataset is available in the Open Science Framework database (https://osf.io/hfpby). We used summative scales with equally weighted components to measure Cultural Relevance, Connectedness to Nature, and Self-Efficacy (Table 1). Response options for all of the components featured a Likert-type scale, in which the choices were, “Strongly Disagree” (“1”) to “Strongly Agree” (“5”). We conducted factor analyses on the questionnaire items comprising each of the three scales. These analyses revealed that each indicator had a high loading on its underlying concept and that the loadings were very similar in magnitude (between 0.63 and 0.78). The values of Cronbach’s Alpha are high for each of the scales (Table 1). (Cronbach’s alpha, the most widely used scale-reliability measure, gauges the internal consistency of the items comprising the scale and varies from zero to one. Higher values indicate better reliability.)

The surveys also included two demographic items (gender and race/ethnicity) as well as three outcome (behavioral) measures. The latter are: (1) “I pay attention to the way the clouds are moving” [Clouds]; (2) “I ignore trash when I see it on the shoreline” [Trash, reverse coded]; (3) “I turn the water off when I’m brushing my teeth” [Water]. Our analyses indicated weak-to-moderate inter-correlations among these items; therefore, we retained each as an individual item. We scaled these responses from 1 to 5, with “1” representing “Never,” and “5” representing “Always.” We reverse-coded the Trash item to maintain consistency among measures.

All measures behave well from a distributional perspective: standard deviations are always much less than means. Bivariate correlations among our variables range from 0.000 to 0.470 in absolute value, indicating no collinearity issues. After listwise deletion of missing data, 98 cases remain for analysis. We chose listwise deletion over multiple imputation for two reasons: First, we lose only 12 cases, so the gain in sample size from multiple imputation would be very small. Second, listwise deletion is, at times, equivalent to multiple imputation and, at times, better than multiple imputation [47].

**Fig 1:**
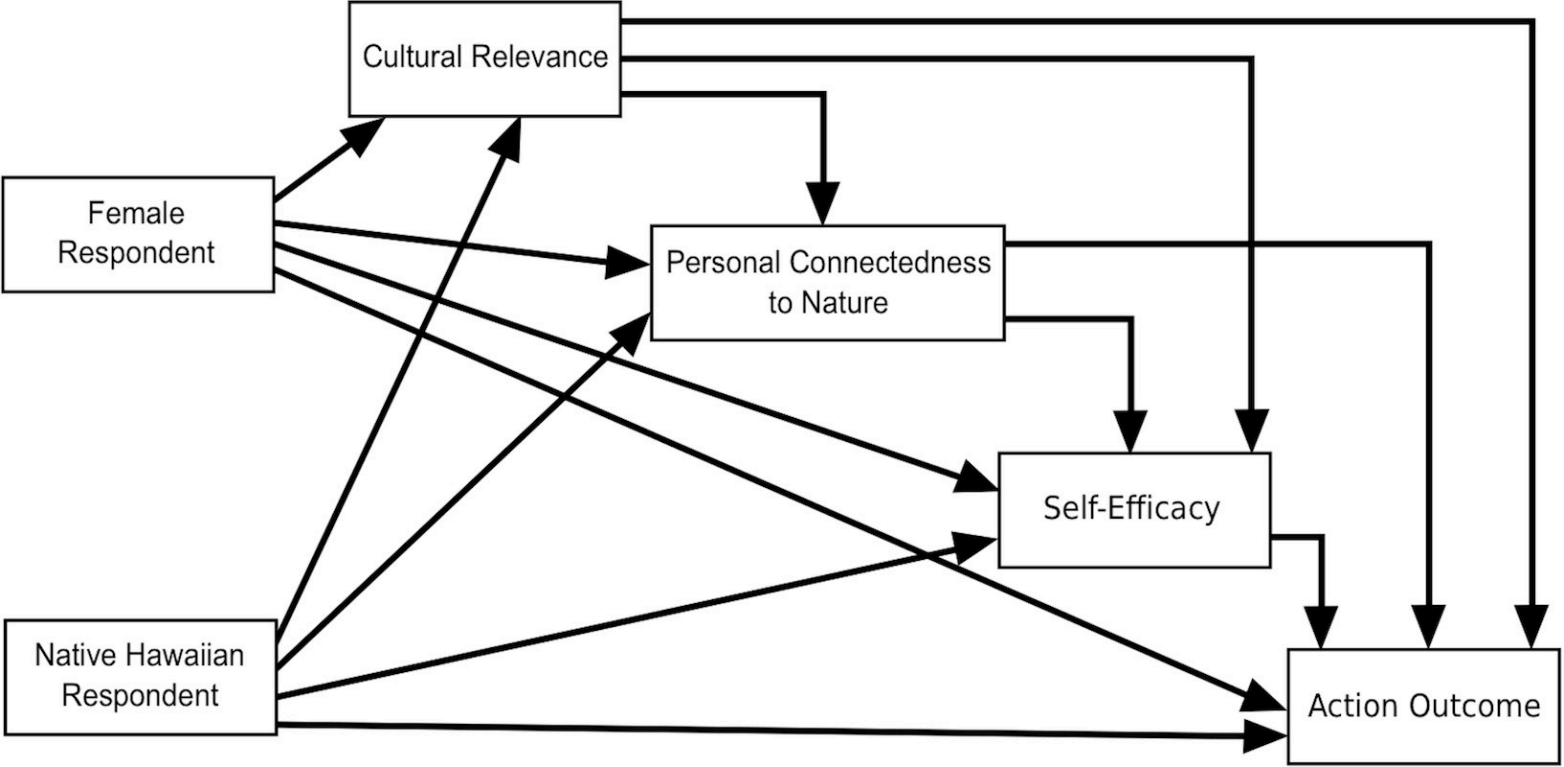
Fully recursive path analysis model.

We estimate the parameters of a fully recursive path model using multivariate Ordinary Least Squares (Fig 1). This means we have six multiple regression equations, with each path arrow in the diagram representing the effect of an independent variable on a dependent variable, controlling for the other independent variables. Thus, the model depicts a set of causal hypotheses and can be read from left to right in the following manner: (1) the demographic variables influence Cultural Relevance; (2) the demographic variables and Cultural Relevance affect Connectedness to Nature; (3) the demographic variables, Cultural Relevance, and Connectedness to Nature affect Self-Efficacy; and (4) all variables in (1) through (3) affect the behavioral variables (Clouds, Trash, and Water).

Given the small sample size, we used a statistical significance level of 0.10 rather than the conventional 0.05 level. Power analysis suggests that a 0.10 significance level allows for balancing Type I and Type II error, and researchers agree that the probabilities of Type I and Type II errors are inversely related [48]. In the model that includes Clouds as the dependent variable, for instance, the average power for the tests of statistical significance for the regression coefficients increases from 0.345 to 0.433 when we raise the alpha level from 0.05 to 0.10. When sample sizes are large, the probabilities of Type I and Type II errors are small, so such adjustments are unnecessary. We did not conduct a priori power analyses, as we did not know what the effect sizes would be. We also did not engage in the dubious practice of post-hoc, observed-power analysis [49]. We present the observed power statistics simply as estimates of the increase in power resulting from a change in the significance level. We do not argue post-hoc for rejecting the non-rejected null hypotheses.

To corroborate the results based on p-values, we also report and discuss the model selection statistic AIC (Akaike Information Criterion), as well as several statistics based on this measure.

## Results

Our sample population included diversity in terms of ethnicity and length of residence in Hawaiʻi. According to our respondents, 44% of student families had lived in Hawaiʻi for more than two generations; 12% had lived in Hawaiʻi for at least 25 years (roughly one generation); and the majority (66%) had lived in Hawaiʻi for students’ entire lives. The students in our sample self-identified as five major ethnicities, with many listing multiple ethnicities: 44.9% identified as Native Hawaiian, 25.5% as Chinese, 37.8% as Filipino, 29.6% as Japanese, and 36.7% as White. (Again, the ethnicity categories that respondents could select were not mutually exclusive.) Those identifying as at least part Native Hawaiian were more prevalent in our sample than in the general population; the population of Hawaiʻi Island at the time of the survey was approximately 46% White alone, 25% Native Hawaiian, 19% Asian, and 10% Other [42]. The difference between the overall island population and our sample population was likely due to a combination of factors with a primary one being that many of the non-Native Hawaiian residents in the part of the island served by the school do not have school-aged children.

Respondents with Native Hawaiian backgrounds were more likely than others to report that Hawaiian culture feels relevant to their lives. (Note that our sample size is too small to examine the effects of membership in other ethnic groups.) On the Cultural Relevance scale, which has an observed range of 11 points, respondents with Native Hawaiian background had scores that were, on average, about 1.2 points higher than those without Native Hawaiian background (Table 2). Respondents with higher cultural relevance ratings exhibited a higher connectedness to nature, enhanced self-efficacy, and a greater likelihood of picking up trash. In addition, respondents who reported a stronger connection to nature were also more likely also to report paying attention to moving clouds. Gender had no statistically significant effect on any dependent variable in our model. Being of Native Hawaiian heritage had no effect on any dependent variable once we controlled for Cultural Relevance, indicating that Cultural Relevance mediated the effect of ethnic identity. None of our variables had a statistically significant effect on turning off the water while brushing one’s teeth (Table 2).

**Table 2:** OLS estimates of the parameters of the fully recursive path model: Unstandardized regression coefficients (p-values for two-tailed t-tests are in parentheses).

			Dependent Variables			
	Cultural Relevance	Connectedness to Nature	Self-Efficacy	Clouds	Trash	Water
Independent Variables						
Female	-.171	-.515	.070	.029	-.007	.310
	(.645)	(.255)	(.874)	(.894)	(.965)	(.186)
Native Hawaiian	1.194	.272	-.388	-.014	.048	-.037
	(.002)	(.568)	(.400)	(.952)	(.766)	(.880)
Cultural Relevance		.579	.595	-.139	-.100	-.002
		(.000)	(.000)	(.057)	(.057)	(.983)
Connectedness to Nature			.042	.132	-.042	.007
			(.672)	(.008)	(.236)	(.900)
Self-Efficacy				.085	-.026	.035
				(.098)	(.477)	(.529)
R-Squared	.099	.227	.228	.103	.129	.024
AIC with Cultural Relevance		160.068	153.631	14.834	-49.579	30.982
AIC without Cultural Relevance	120.884	178.291	176.217	12.86	-40.806	29.129
Change in AIC when Cultural						
Relevance Added to Model		-18.223	-22.586	1.974	-8.773	1.853
Delta AIC for Model with Cultural Relevance		0	0	1.974	0	1.853
AIC Weight for Model with Cultural Relevance		1.000	1.000	.272	.988	.284
AIC Weight for Model without Cultural Relevance		.000	.000	.728	.012	.716

In contrast to previous research, our multivariate models did not detect an effect of self-efficacy on either typical Western pro-environmental outcome: picking up trash or turning off water. This may be, in part, because omitting a measure of culture in previous models created a misspecification. When, for example, we examined the bivariate association between self-efficacy and picking up trash, self-efficacy had a statistically significant effect. The same is true for the bivariate relationship between connectedness to nature and self-efficacy. That the measure of cultural relevance we developed played a prominent role in our models suggests the importance of this previously omitted variable. The effects of cultural relevance are not only statistically significant, but they are also strong in magnitude: the standardized regression coefficients are about 0.45 in the connectedness-to-nature and self-efficacy equations, and about 0.23 in the trash equation. These rather-large standardized regression coefficients are consistent with other statistics presented in the table. Most importantly, the model selection statistic AIC (Akaike Information Criterion) shows a preference for including cultural relevance in the connectedness-to-nature, self-efficacy, and trash models (see Table 2). AIC values decline by about 18, 23, and 9, respectively, when cultural relevance is added to these models, and the AIC weights indicate that, given the data, conditional probabilities of the models are very high.

Our findings suggest a complex relationship between cultural relevance, self-efficacy, and cloud observation. As mentioned above, cultural relevance was positively associated with self-efficacy; self-efficacy, in turn, was positively associated with cloud observation. One interpretation of those relationships, suggested by our models, is that cultural relevance positively yet indirectly affects cloud watching, via self-efficacy.

## Discussion

We found that local cultural relevance, a construct rarely investigated in previous environmental psychological research, was an important element of multiple models. Specifically, our cultural relevance construct significantly related to connectedness to nature, self-efficacy, and picking up trash (a common measure of pro-environmental behavior). Notably, when we omitted cultural relevance, we found statistically significant relationships similar to those in past studies that do not account for culture, i.e., between self-efficacy and a widely accepted measure of pro-environmental behavior (in this case, picking up trash). Our results are, thus, consistent with past findings: had we not included cultural relevance measures, our results would confirm past findings. The strong predictive power of our cultural relevance variable, however, suggests that existing connectedness-to-nature, self-efficacy, and behavior studies, while informative and valuable, may omit an important construct.

Scholars from a variety of fields have drawn attention to the lack of consideration of culture in environmental endeavors. Environmental education researchers, for instance, have questioned the narrow focus on a few limited indicators and outcomes, such as connectedness to nature and self-efficacy (e.g. [50]). They theorize that cultural identity and relevance play a much more important role than is often recognized, and they argue for a broadening of environmental education by considering culturally relevant pedagogies [51]. Those arguments closely relate to environmental justice concerns, and other scholars discuss the urgent need to address such concerns in environmental work more broadly (i.e., not only in environmental education). One important suggestion is to incorporate cultural histories in discussions of the environment, wilderness, and related concepts [52–54]. Scholars from disciplines including ecology, psychology, sociology, anthropology, and education simultaneously critique the traditional colonialist way of viewing the land (i.e., anthropocentric approaches that portray land as a resource to be dominated, controlled, and exploited) and advocate for valuing multiple ways of knowing and learning about land and ecosystems, as well as the entities that comprise them [30,50,55,56].

These scholars and others suggests that the lack of attention to cultural heterogeneity may be intertwined with the dramatic underrepresentation of people of color in the environmental field as well as the closely related issue of current approaches to environmental programming being less effective and appropriate for culturally diverse populations [52,53,57]. Our findings provide a case where this may be relevant. Given the centrality of land and water to Hawaiian culture, addressing pro-environmental attitudes and behaviors in Hawaiʻi may relate to cultivating knowledge of and respect for Hawaiian culture, rather than focusing primarily on the relationship between nature connectedness and self-efficacy. Numerous Hawaiian initiatives take this approach and work to raise awareness and understanding of Hawaiian culture with multiple desired outcomes, including a focus on environmental stewardship. The varied programs of the Kamehameha Schools Trust (KS) provide one well-known example: KS administers programs that offer education on Native Hawaiian cosmovision, practices, and intricate relationships with place (http://www.ksbe.edu/communityeducation/).

Similarly, environmentally focused nonprofit organizations and philanthropic institutions across the United States pursue and encourage culturally sensitive approaches in varied contexts. Those initiatives recognize the repeated findings of notable variation in the type and extent of environmental concern among people of different backgrounds (e.g. [5]). Outdoor Afro, for example, is a national network that “celebrates and inspires African American connections and leadership in nature” (http://www.outdoorafro.com/about/). The Doris Duke Conservation Scholars Program trains and inspires future conservation professionals with extensive attention to cultural diversity (http://uwconservationscholars.org/). Red Bike & Green is “a community-building collective of Black urban cyclists seeking to improve the physical and mental health, economy and local environment of African Americans by creating a relevant and sustainable Black bike culture” (http://www.redbikeandgreen.com/). As a final example, the NSF-funded iWISE (Indigenous Worldviews in Informal Science) initiative develops resources and hosts workshops designed to explore the intersection of indigenous ways of knowing with STEM education; much of their programming involves environmental issues (http://iwiseconference.org).

These initiatives, along with others, provide evidence of the environmental field seeking to move beyond traditional definitions and include multiple ways of knowing. In essence, they reflect conversations that incorporate and consider culture as a dynamic, multifaceted variable. Yet further progress is needed: Many of the organizations described were formed with the intention of addressing a lack of diversity in the environmental field; their work suggests that, despite good intentions, cultural pluralism within this realm remains a challenge.

Our findings align with prior critiques and efforts in the environmental movement. We suggest, as does much of that previous work, some potentially helpful considerations for initiatives focused on environmental attitudes, knowledge, values, and behaviors. Perhaps most importantly, the statistical importance of our cultural relevance variable suggests that omitting considerations of culture in program design may lead to a focus on elements of program design that are less impactful or relevant. Once an initiative or institutions commits to and recognizes the importance of addressing cultural pluralism, the question then becomes how to effectively and meaningfully acknowledge and incorporate elements of cultural identity into conservation efforts.

We suggest that research on, and practices related to, culturally relevant pedagogy from the education field may offer guidance. A few studies have investigated interventions developed with particular attention to culturally relevant practices and found positive results in program outcomes, such as higher engagement by Latinx environmental educators and greater participation in recycling programs [58,59]. Such efforts, which can provide specific examples and guidance, may also demonstrate how attention to cultural identities can positively influence program processes and outcomes.

Finally, some environmental behavior research finds gender-related differences in engagement in pro-environmental behavior. Those findings, which suggest that women are often more likely than men to engage in pro-environmental behavior, have been relatively consistent across age, decade, and nation [60,61]. Some may therefore find the lack of significance of gender-based differences in our data to be puzzling. Overall, however, researchers have found the magnitude of the gender effect to be small in previous research [62], and our sample may not be large enough to reveal that effect. In addition, the lack of effect in our data may relate to our participants’ young age. Gender and age may interact, and the social conventions that may explain higher levels of pro-environmental behavior among adult women perhaps are less solidified. Lastly, in Hawaiʻi, respect for ecosystems is an important part of the cultural tradition *writ large* and, as such, may negate gender effects. One interesting future comparison would be to consider gender effects in similar societies with a study like ours.

Our anomalous finding of a negative relationship between cultural relevance and the cloud observation behavior—the item that our partners designed to indicate a locally culturally relevant environmental behavior—is also confusing. This negative relationship is, however, likely a statistical artifact, which can be seen most easily seen by considering the sign of the numerator for the partial correlation coefficient (the denominator is always positive): Correlation(clouds&culture, controlling for connection.2.nature) = Correlation(clouds & culture)—Correlation(clouds& connection.2.nature) * Correlation(culture& connection.2.nature). In our data, the bivariate correlation between clouds and culture is zero, while the correlations between both clouds and nature and culture and nature are positive. This means that the partial correlation must be negative. The findings that children with a higher connectedness to nature are more likely to view the clouds, and that children with a higher attachment to local culture are more likely to exhibit a connectedness to nature, are logical and expected.

The lack of a bivariate correlation between clouds and culture is interesting from a substantive point of view. One potential explanation is that our cultural relevance measure targets different aspects of local culture than does the specific cloud observation item: children for whom local culture in general is relevant may not be aware that cloud observation is, for some people at least, part of that culture. Encouraging Native Hawaiian practices, such as monitoring clouds for the environmental information they provide, is an aspirational goal of culturally relevant environmental education in Hawaiʻi. Our data suggest that, at least for this eighth-grade sample, the connection between cloud observation and Native Hawaiian culture may not be widely understood. Regardless, this nuanced relationship warrants further study, including potential qualitative work exploring how students think about local culture, including specific elements of local culture including cloud observation, and how these phenomena relate to other variables of interest, such as young people’s feelings of self-efficacy.

### Limitations, delimitations, and future research

Like all studies, ours has limitations. We pursued this study by fusing philosophical orientations, and relatedly research constructs, from multiple disciplines, which can bring both strengths and weaknesses to research design and implementation. Our interdisciplinary approach draws on notions of culture from anthropology; ideas about influences on pro-environmental behavior from psychology and sociology; and psychometric tools common in psychology and sociology, facilitating application of various perspectives to the complex phenomena under consideration.

In terms of challenges and limitations, by combining various approaches and epistemological lenses, we risk confusion around language and perceptions of “short-changing” some concepts. Anthropologists, for example, may justifiably protest our over-simplification of “cultural relevance.” We do not purport to fully and accurately encapsulate a deep meaning or relevance of culture, especially in Hawaiʻi, which is a place where notions of both culture and relevance are particularly rich, varied, and—at times—contentious. Researchers focusing on the role of culture (in some form) in environmental management (e.g [63]) characterize elusive notions of “culture” in more nuanced ways. Rarely, however, do such studies use psychometric items that facilitate quantitative and relational comparisons among constructs, such as those pursued in this study. Psychologists, similarly, may protest the sparseness of our self-efficacy measures. Self-efficacy phenomena, and related constructs, are multi-step and the subject of scores of studies [64,65]. Our instrument characterizes self-efficacy using four items, thus reducing its complexity to make measurement manageable.

Additional limitations include our small sample size, which limits generalizability of results, and the potential bias introduced by different teachers administering the survey. One final limitation relates to the aphorism, “all models are wrong, and some are useful.” Although our survey items do not fully characterize our participants’ complex relationships with the local Hawaiian culture or the multi-dimensional nature of self-efficacy, we suggest that our model may nevertheless be useful. Because models are approximate reflections of the world, they are always imperfect.

### Conclusion

Our cultural relevance index correlates with connection to nature, self-efficacy, and environmental behavior, suggesting a place for more emphasis on cultural pluralism in environmental work. Yet our results are far from definitive. They are a first step that indicate the necessity of further exploring the essentially limitless variants of culture and how those may interact with environmental attitudes and behaviors. Building on our results, an important aspect of future work would be to engage more deeply with the idea that culture reflects multiplicity. One might do so through collaborating with and researching programs that include multiple ways of knowing, different definitions of “environment,” and a range of actions that demonstrate environmental caring. Hundreds of environmental initiatives might provide fodder for such a broadening and deepening of the roles that culture plays in environmental issues [54,66]. This attention to culture(s) may be the defining feature of the next era of the environmental movement, which will embrace an array of perspectives and voices growing out of and in response to cultural differences, in a variety of communities, at a range of scales.

## Supporting information

S1 File Provides the survey instrument distributed to students. (PDF)

## References

[1] Kollmuss A, Agyeman J. Mind the gap: why do people act environmentally and what are the barriers to pro-environmental behavior? Environmental Education Research. 2002;8: 239–260.

[2] Axon S. “Keeping the ball rolling”: Addressing the enablers of, and barriers to, sustainable lifestyles. Journal of Environmental Psychology. 2017;52: 11–25. 10.1016/j.jenvp.2017.05.002

[3] Bamberg S, Möser G. Twenty years after Hines, Hungerford, and Tomera: A new meta-analysis of psycho-social determinants of pro-environmental behaviour. J Environ Psychol. 2007;27: 14–25.

[4] Shove E. Comfort, cleanliness and convenience: the social organization of normality Oxford: Berg; 2003.

[5] Macias T. Ecological Assimilation: Race, Ethnicity, and the Inverted Gap of Environmental Concern. Society and Natural Resources. 2016;29: 3–19.

[6] Geertz C. The Interpretation of Cultures. New York: Basic Books; 1973.

[7] Olli E, Grendstad G, Wollebaek D. Correlates of environmental behaviors bringing back social context. Environment and behavior. 2001;33: 181–208.

[8] Theodori GL. Community Attachment, Satisfaction, and Action. Journal of the Community Development Society. 2004;35: 73–86. 10.1080/15575330409490133

[9] Markus HR. What moves people to action? Culture and motivation. Current Opinion in Psychology. 2016;8: 161–166. 10.1016/j.copsyc.2015.10.028 29506793

[10] Lehman DR, Chiu C, Schaller M. Psychology and Culture. Annu Rev Psychol. 2004;55: 689–714. 10.1146/annurev.psych.55.090902.141927 14744231

[11] Medin DL, Bang M. Who’s asking?: Native science, Western science, and science education The MIT Press; 2013.

[12] Restall B, Conrad E. A literature review of connectedness to nature and its potential for environmental management. Journal of Environmental Management. 2015;159: 264–278. 10.1016/j.jenvman.2015.05.022 26087657

[13] Tam K-P. Concepts and measures related to connection to nature: Similarities and differences. Journal of Environmental Psychology. 2013;34: 64–78. 10.1016/j.jenvp.2013.01.004

[14] Mayer FS, Frantz CMP. The connectedness to nature scale: A measure of individuals’ feeling in community with nature. Journal of Environmental Psychology. 2004;24: 503–515.

[15] Schultz PW, Tabanico J. Self, identity, and the natural environment: Exploring implicit connections with nature. Journal of Applied Social Psychology. 2007;37: 1219–1247.

[16] Geng L, Xu J, Ye L, Zhou W, Zhou K. Connections with Nature and Environmental Behaviors. Friese M, editor. PLoS ONE. 2015;10: e0127247 10.1371/journal.pone.0127247 25985075 PMC4436134

[17] Mayer FS, Frantz CM, Bruehlman-Senecal E, Dolliver K. Why Is Nature Beneficial?: The Role of Connectedness to Nature. Environment and Behavior. 2008;41: 607–643. 10.1177/0013916508319745

[18] Gosling E, Williams KJ. Connectedness to nature, place attachment and conservation behaviour: Testing connectedness theory among farmers. Journal of Environmental Psychology. 2010;30: 298–304.

[19] Axelrod LJ, Lehman DR. Responding to environmental concerns: What factors guide individual action? Journal of Environmental Psychology. 1993;13: 149–159. 10.1016/S0272-4944(05)80147-1

[20] Bandura A. Self-efficacy: toward a unifying theory of behavioral change. Psychological review. 1977;84: 191 847061 10.1037//0033-295x.84.2.191

[21] Allen JB, Ferrand JL. Environmental Locus of Control, Sympathy, and Proenvironmental Behavior A Test of Geller’s Actively Caring Hypothesis. Environment and Behavior. 1999;31: 338–353. 10.1177/00139169921972137

[22] Kaplan S, Kaplan R. Health, supportive environments, and the reasonable person model. American Journal of Public Health. 2003;93. 10.2105/ajph.93.9.1484 PMC1447997 12948967

[23] Ajzen I. From Intentions to Actions: A theory of planned behavior In: Kuhl J, Beckmann J, editors. Action control: From cognition to behavior. Berlin Heidelberg: Springer-Verlag; 1985 pp. 11–39.

[24] Ajzen I. Perceived Behavioral Control, Self-Efficacy, Locus of Control, and the Theory of Planned Behavior1. Journal of Applied Social Psychology. 2002;32: 665–683. 10.1111/j.1559-1816.2002.tb00236.x

[25] Tabernero C, Hernández B. Self-Efficacy and Intrinsic Motivation Guiding Environmental Behavior. Environment and Behavior. 2011;43: 658–675. 10.1177/0013916510379759

[26] Wu H, Mweemba L. Environmental self-efficacy, attitude and behavior among small scale farmers in Zambia. Environment, Development and Sustainability. 2010;12: 727–744. 10.1007/s10668-009-9221-4

[27] McCarty JA, Shrum LJ. The influence of individualism, collectivism, and locus of control on environmental beliefs and behavior. J Public Policy Mark. 2001;20: 93–104.

[28] Gifford R, Nilsson A. Personal and social factors that influence pro-environmental concern and behaviour: A review. Int J Psychol. 2014;49: 141–157. 10.1002/ijop.12034 24821503

[29] Nisbett RE, Peng K, Choi I, Norenzayan A. Culture and systems of thought: holistic versus analytic cognition. Psychol Rev. 2001;108: 291–310. 11381831 10.1037/0033-295x.108.2.291

[30] Bang M, Medin DL, Atran S. Cultural mosaics and mental models of nature. PNAS. 2007;104: 13868–13874. 10.1073/pnas.0706627104 17715299 PMC1955807

[31] Washinawatok K, Rasmussen C, Bang M, Medin D, Woodring J, Waxman S, et al Children’s Play with a Forest Diorama as a Window into Ecological Cognition. Journal of Cognition and Development. 2017;18: 617–632. 10.1080/15248372.2017.1392306

[32] Unsworth SJ, Levin W, Bang M, Washinawatok K, Waxman SR, Medin DL. Cultural Differences in Children’s Ecological Reasoning and Psychological Closeness to Nature: Evidence from Menominee and European American Children. Journal of Cognition & Culture. 2012;12: 17–29. 10.1163/156853712X633901

[33] Turner NJ. The Earth’s Blanket: traditional teachings for sustainable living. Douglas & McIntyre; 2005.

[34] McGregor DP. An introduction to the Hoa’äina and their rights. Hawaiian Journal of History. 1996;30: 1–27.

[35] Tuck E, McKenzie M, McCoy K. Land education: Indigenous, post-colonial, and decolonizing perspectives on place and environmental education research. Environmental Education Research. 2014;20: 1–23. 10.1080/13504622.2013.877708

[36] Taylor B. Dark green religion: Nature spirituality and the planetary future. Berkeley, California, USA: University of California Press; 2009.

[37] Vickery J, Hunter LM. Native Americans: Where in Environmental Justice Research? Society & Natural Resources. 2016;29: 36–52. 10.1080/08941920.2015.1045644 27103758 PMC4835033

[38] Nadasdy P. The gift in the animal: The ontology of hunting and human–animal sociality. American Ethnologist. 2007;34: 25–43.

[39] Pascua P, McMillen H, Ticktin T, Vaughan M, Winter KB. Beyond services: A process and framework to incorporate cultural, genealogical, place-based, and indigenous relationships in ecosystem service assessments. Ecosystem Services. 2017;26: 465–475. 10.1016/j.ecoser.2017.03.012

[40] The Kumulipo. Lexington, KY: Forgotten Books; 2008.

[41] Stewart F, editor. Wao Akua: Sacred Source of Life. Honolulu, HI: Hawaii Department of Land and Natural Resoures/Division of Forestry and Wildlife; 2003.

[42] U.S. Census Bureau. 2006–2010 American Community Survey. 2011.

[43] Osorio JKK, Howes C, editors. The Value of Hawaii: Knowing the Past, Shaping the Future. Honolulu, HI: University of Hawai’i Press; 2010.

[44] Perrin JL, Benassi VA. The connectedness to nature scale: A measure of emotional connection to nature? Journal of Environmental Psychology. 2009;29: 434–440. 10.1016/j.jenvp.2009.03.003

[45] Stern MJ, Powell RB, Ardoin NM. Evaluating a constructivist and culturally responsive approach to environmental education for diverse audiences. The Journal of Environmental Education. 2010;42: 109–122.

[46] Pukui MK. ‘Ōlelo No’eau: Hawaiian Proverbs & Poetical Sayings. Honolulu, HI: Bishop Museum Press; 1997.

[47] Allison PD. Missing Data. Thousand Oaks, CA: SAGE Publications, Inc; 2002.

[48] Cohen J, Cohen P. Applied Multiple Regression/Correlation Analysis for the Behavioral Sciences.: Hillsdale, NJ: Erlbaum Associates; 1983.

[49] Hoenig JM, Heisey DM. The abuse of power: The pervasive fallacy of power calculations for data analysis. The American Statistician. 2001;55: 19–24.

[50] McKenzie M. The ‘post‐post period’ and environmental education research. Environmental Education Research. 2005;11: 401–412. 10.1080/13504620500169361

[51] Nordström HK. Environmental Education and Multicultural Education–Too Close to Be Separate? International Research in Geographical and Environmental Education. 2008;17: 131–145. 10.1080/10382040802148604

[52] Finney C. Black Faces, White Spaces: Reimagining the Relationship of African Americans to the Great Outdoors 1 edition Chapel Hill: The University of North Carolina Press; 2014.

[53] Taylor DE. The Rise of the American Conservation Movement: Power, Privilege, and Environmental Protection. Durham, N.C: Duke University Press; 2016.

[54] Gould RK, Phukan I, Mendoza ME, Ardoin NM, Panikkar B. Seizing opportunities to diversify conservation. CONSERVATION LETTERS. 2018; e12431 10.1111/conl.12431

[55] Lotz-Sisitka H. Changing Social Imaginaries, Multiplicities and “One Sole World”: Reading Scandinavian Environmental and Sustainability Education Research Papers with Badiou and Taylor at Hand. Environmental Education Research. 2010;16: 133–142.

[56] Berkes F. Evolution of co-management: Role of knowledge generation, bridging organizations and social learning. Journal of Environmental Management. 2009;90: 1692–1702. 10.1016/j.jenvman.2008.12.001 19110363

[57] Taylor DE. The State of Diversity in Environmental Organizations. Green 2.0; 2014.

[58] Arreguín-Anderson MG, Kennedy KD. Deliberate Language Planning in Environmental Education: A CRT/LatCrit Perspective. The Journal of Environmental Education. 2013;44: 1–15. 10.1080/00958964.2012.665098

[59] Bogin R. Reciclaje de crecimiento (growing recycling). Resource Recycling. 2009;28: 28–31.

[60] Yates A, Luo Y, Mobley C, Shealy E. Changes in public and private environmentally responsible behaviors by gender: findings from the 1994 and 2010 general social survey. Sociological Inquiry. 2015;85: 503–531.

[61] Strapko N, Hempel L, MacIlroy K, Smith K. Gender Differences in Environmental Concern: Reevaluating Gender Socialization. Society & Natural Resources. 2016;29: 1015–1031.

[62] Milfont TL, Sibley CG. Empathic and social dominance orientations help explain gender differences in environmentalism: A one-year Bayesian mediation analysis. Personality and Individual Differences. 2016;90: 85–88.

[63] Christianson A, Mcgee TK, L’Hirondelle L. The Influence of Culture on Wildfire Mitigation at Peavine Métis Settlement, Alberta, Canada. Society & Natural Resources. 2014;27: 931–947. 10.1080/08941920.2014.905886

[64] Clayton S, Prévot A-C, Germain L, Saint-Jalme M. Public Support for Biodiversity After a Zoo Visit: Environmental Concern, Conservation Knowledge, and Self-Efficacy. Curator. 2017;60: 87–100. 10.1111/cura.12188

[65] Estrada M, Schultz PW, Silva-Send N, Boudrias MA. The Role of Social Influences on Pro-Environment Behaviors in the San Diego Region. Journal of Urban Health. 2017; 1–10. 10.1007/s11524-016-0118-x 28265806 PMC5391335

[66] Hawken P. Blessed unrest: How the largest movement in the world came into being, and why no one saw it coming. Penguin; 2007.

