# Supplementary material for: The importance of culture in predicting environmental behavior in middle school students on Hawaiʻi Island
Source: PLoS One. 2018 Nov 12;13(11):e0207087. doi: 10.1371/journal.pone.0207087 (PMC6231625; doi:10.1371/journal.pone.0207087)
Supplement: S1 File — (PDF) [file pone.0207087.s001.pdf]

***Instructions* : Read each question carefully and respond as best you can. When you're finished, place your completed sheet into the folder on the teacher's desk. Do not talk to your neighbors while filling out the survey.**

Are you female or male? *Please circle one.* **Female** **Male**

Write your initials here: ________________

Write the month, day and year of your birthday: **Month:** _______ **Day:** _______ **Year:** ______

Please answer each of these questions in terms of the way you generally feel. There are no right or wrong answers. Using the following scale, next to each question simply circle as honestly as you can.

I feel that all living things in this world are connected, and I am a part of that.	**Strongly Disagree**	**Disagree**	**Neutral**	**Agree**	**Strongly Agree**
The choices I make today can change my entire life.	**Strongly Disagree**	**Disagree**	**Neutral**	**Agree**	**Strongly Agree**
I learn a lot when I am outdoors.	**Strongly Disagree**	**Disagree**	**Neutral**	**Agree**	**Strongly Agree**
I often feel a strong connection to nature.	**Strongly Disagree**	**Disagree**	**Neutral**	**Agree**	**Strongly Agree**
I feel it's important to learn about traditional cultural practices in my community.	**Strongly Disagree**	**Disagree**	**Neutral**	**Agree**	**Strongly Agree**
I identify strongly with Kona's shorelines.	**Strongly Disagree**	**Disagree**	**Neutral**	**Agree**	**Strongly Agree**
I see myself as a part of the greater circle of life.	**Strongly Disagree**	**Disagree**	**Neutral**	**Agree**	**Strongly Agree**
I feel comfortable applying ancient Hawaiian wisdom.	**Strongly Disagree**	**Disagree**	**Neutral**	**Agree**	**Strongly Agree**
I think of nature as a family that I belong to.	**Strongly Disagree**	**Disagree**	**Neutral**	**Agree**	**Strongly Agree**
My actions impact the environment.	**Strongly Disagree**	**Disagree**	**Neutral**	**Agree**	**Strongly Agree**
When I need help, my culture supports me.	**Strongly Disagree**	**Disagree**	**Neutral**	**Agree**	**Strongly Agree**
Kona's shorelines are very special to me.	**Strongly Disagree**	**Disagree**	**Neutral**	**Agree**	**Strongly Agree**
I believe that understanding the history of my community makes me a stronger person.	**Strongly Disagree**	**Disagree**	**Neutral**	**Agree**	**Strongly Agree**
Humans are more important than plants and animals.	**Strongly Disagree**	**Disagree**	**Neutral**	**Agree**	**Strongly Agree**
I don't feel part of nature.	**Strongly Disagree**	**Disagree**	**Neutral**	**Agree**	**Strongly Agree**
My school is a dangerous place.	**Strongly Disagree**	**Disagree**	**Neutral**	**Agree**	**Strongly Agree**
I can be a good leader.	**Strongly Disagree**	**Disagree**	**Neutral**	**Agree**	**Strongly Agree**

I feel related to animals and plants.	Strongly Disagree	Disagree	Neutral	Agree	Strongly Agree
I feel like Kona's shorelines are a part of me.	Strongly Disagree	Disagree	Neutral	Agree	Strongly Agree
I have the power to help protect the environment.	Strongly Disagree	Disagree	Neutral	Agree	Strongly Agree
I feel I belong to the Earth and the Earth belongs to me.	Strongly Disagree	Disagree	Neutral	Agree	Strongly Agree
I have the power to help protect my culture.	Strongly Disagree	Disagree	Neutral	Agree	Strongly Agree
Like a tree in the forest, I feel I belong to nature.	Strongly Disagree	Disagree	Neutral	Agree	Strongly Agree
I enjoy learning.	Strongly Disagree	Disagree	Neutral	Agree	Strongly Agree
I can make a change in my community.	Strongly Disagree	Disagree	Neutral	Agree	Strongly Agree
There is something that every living thing shares.	Strongly Disagree	Disagree	Neutral	Agree	Strongly Agree

Questions 15-18. Please check the box that best describes your level of interest in each of the following activities.

Learning about new subjects in school	Not interested	Hardly interested	A little interested	Pretty interested	Very interested
Learning how to understand the environment	Not interested	Hardly interested	A little interested	Pretty interested	Very interested
Going to college	Not interested	Hardly interested	A little interested	Pretty interested	Very interested
Working to make my community a better place	Not interested	Hardly interested	A little interested	Pretty interested	Very interested

Questions 21-29. How often do you do the following things?

I talk to my family or friends outside of school about what I've learned.	Never	Hardly ever	Sometimes	Often (or usually)	Always
I ignore trash when I see it on the shoreline.	Never	Hardly ever	Sometimes	Often (or usually)	Always
I work as a volunteer in my community.	Never	Hardly ever	Sometimes	Often (or usually)	Always
I research things that I am curious about.	Never	Hardly ever	Sometimes	Often (or usually)	Always
I talk to my friends about choices that will have impacts after we're gone (like, in 200 years).	Never	Hardly ever	Sometimes	Often (or usually)	Always
I turn off the water when I'm brushing my teeth.	Never	Hardly ever	Sometimes	Often (or usually)	Always
I pay attention to the way clouds are moving.	Never	Hardly ever	Sometimes	Often (or usually)	Always

About how many times have you littered in the past 3 months?

____ Not at all ____ Once or twice ____ 3 to 5 times ____ 6 to 10 times ____ More than 10 times

For the last two questions, please write a few sentences in response to each question:

What does ‘Hawaiian’ mean?

When, if ever, is it appropriate to cut down a 300-year-old tree?

Thank you very much for your participation.

Appendix – Survey Instrument

The survey instrument distributed to students is provided on the following three pages.

It is presented in the format provided to students.
